# Paulus Ægineta: Four Examples of His Skill

**Published:** 1912-11-09

**Authors:** 


### MEMOS. FROM THE MASTERS.

### Paulus Ægineta: Four Examples of His Skill.

Whatever classification we adopt there will be great difference in the treatment of the varieties of erysipelas. This, indeed, must be recognised as being a most dangerous disease, especially when it attacks the head, and if not actively treated from the first, it will cause swelling and kill the victims by suffocating them. Begin then by opening a vein; choose the large vein on the patient's arm, but if that does not stand out, select any prominent vein. If venesection is, through some reason, contra-indicated, recourse must be had to purging by chologogues, or strong clysters may be administered. Locally rub the reddened parts with cooling things, keeping on them constantly moistened sponges that are kept cool by the evaporation of the moisture. The following are useful in this connection: decoctions of house leek, purselain, fleawort, marsh lentil, endive, gourd, nightshade, henbane, and horned poppy. Make a cerate of white wax mixed with four parts of good oil from unripe olives, scented with rose leaves, and add vinegar, thin and transparent, and put this on. Other cerates may be used, of which a good one is: White wax 4 oz., oil of rose (*i.e.* olive oil scented with rose) 3 oz., six eggs and mural pellitory 4 oz. When the inflammation ceases or becomes chronic, before lividity ensues, apply barley-meal poultices. (Lib. iv. sect. xxi.)

Cancer may affect any part of the body. It takes root in the eyes or the uterus, indeed, in any part; but it is most frequently noticed in the breasts of women, these parts being lax and thereby more readily admitting the thick humours which occasion the disease. Cancers are formed by overheated black bile; when this is particularly biting they ulcerate and necrose. They are darker and harder than phlegmons, but have not the same heat. The veins around them are stretched and overfilled, so that they appear to stand out like the feet and legs of the crab. So, indeed, did the disease get its name of The Crab (Cancer), although there are that say it is so called because it adheres firmly even as a crab does to any part that it has laid hold of. Owing to the thickness of the humours it is an incurable malady, neither to be repelled nor remedied. (Lib. iv. sect. xxvii.)

#### A Diagnostic Test for Hydrophobia.

By the following experiment you may find out if the bite has been inflicted by a mad dog or not. Pound walnuts into a paste, which apply to the open wound; the next day take off this cataplasm and give it to a fowl to eat. If hunger compels him to take it, then observe what follows carefully, for if the dog that bit the patient was mad, the fowl will die the next day, but if the dog was healthy then the fowl will live and come to no harm. If the fowl, then, dies, hasten to reopen the wound and keep it so, while if the animal lives it will be permissible to allow the wound to close and cicatricise. (Lib. v.)

#### A Prescription "for Those Beaten with Whips."

Take ceruse and litharge in equal parts, and wax four parts, and mix with rose oil. Apply this mixture as a plaster, using, on the first day, merely an application of oil to the bruises. Another useful application is made as follows: one drachm each of saffron and of tragacanth, which unite with the whole of one egg, shelled, and beat up. But of all remedies this is the best, curing the soonest, even in a day and a night—namely, the hide of a sheep newly taken off, and applied while yet warm.

